# Acute Effects of an Energy Drink on Myocardial Function Assessed by Conventional Echo-Doppler Analysis and by Speckle Tracking Echocardiography on Young Healthy Subjects

**DOI:** 10.1155/2013/646703

**Published:** 2013-11-10

**Authors:** Daniele Menci, Francesca Maria Righini, Matteo Cameli, Matteo Lisi, Susanna Benincasa, Marta Focardi, Sergio Mondillo

**Affiliations:** Department of Cardiovascular Diseases, University of Siena, Viale Bracci, 53100 Siena, Italy

## Abstract

*Purpose*. Previous studies have underlined the effects of the energy drinks containing caffeine end taurine on the cardiovascular system. The aim of this study was to determine acute changes on echocardiographic parameters assessed by conventional echo-Doppler analysis and by speckle tracking echocardiography after the consumption of an energy drink in a young healthy population. *Methods*. measurement of blood pressure, electrocardiographic, and echocardiographic examination were performed on 35 healthy subjects (mean age 25 ± 2 years, 16 men), at baseline and one hour after the consumption of a body surface area indexed amount of an energy drink (168 mL/m^2^) containing caffeine (0.03%) and taurine (0.4%). *Results*. The analysis of left ventricular function showed a significant increase of mean relative values of MAPSE (+11%; *P* < 0.001), global longitudinal strain (+10%, *P* = 0.004), and left ventricular twisting (+22%, *P* < 0.0001) in respect to baseline. Also, right ventricular function parameters appeared significantly increased after energy drink consumption, as TAPSE (+15%, *P* < 0.0001), global, and free wall right ventricular longitudinal strain (+8%, *P* = 0.001; +5%, *P* = 0.1, resp.). *Conclusion*. In conclusion, the consumption of the ED in our population showed a significant increase of right and left ventricular myocardial function, suggesting a possible positive inotropic effect related to the substances contained therein.

## 1. Introduction

 Energy drinks (ED) have increased in popularity among adolescents and young adults, because of the possible ergogenic effects [1–4] and the improvement of cognitive performance due to some ingredients found in these beverages [5, 6], in particular caffeine, taurine, glucuronolactone, and glucose. So far, little evidence exists regarding acute effects of the ED on cardiac function. The most important cardiovascular effects of caffeine in acute settings are the increase of blood pressure and circulating concentration of norepinephrine, the increase of arterial stiffness, and the impairment of endothelium-dependent vasodilation [7].

Taurine (aminoethane sulfonic acid) is a ubiquitous compound found in very high concentrations in heart and muscle. It has been demonstrated that it has a role in the control mechanism of myocardial contractility and studies in animal models have shown that the lack of taurine induces the onset of dilated cardiomyopathy; the beneficial effect of taurine on heart failure was also reported [8]. Moreover, it has been demonstrated that the use of both taurine and caffeine is able to determine a reduction of the effects of caffeine, especially concerning heart rate modifications [9]. Regarding the cardiac effects, an echocardiographic study, concerning the influence of a drink containing taurine and caffeine, performed on healthy subjects, has demonstrated an improvement of left ventricular contractility about forty minutes after taking it [10]. 

The aim of this study was to investigate whether taking a taurine and caffeine containing energy drink determines acute changes in myocardial function assessed by conventional echo-Doppler analysis and by speckle tracking echocardiography (STE), a new technique for assessing myocardial function [11]. 

## 2. Materials and Methods

### 2.1. Study Population

The study group included 35 healthy young volunteers (mean age 25 ± 2 years, 16 men). All subjects had unremarkable history and normal findings at physical examination, electrocardiogram, and echocardiography. None of them was taking any drugs. Ten of them were smokers. Eight subjects were usual consumers of energy drinks (no more than a can a week). Only 7 of them practiced sports. Characteristics of the study population are reported in Table 1. 

### 2.2. Study Protocol

All participants were asked to abstain from smoking, coffee, and other food or beverages containing caffeine for at least 12 hours before the examinations. All the examinations were carried out in the afternoon between meals. Baseline clinical, blood pressure, ECG, and echocardiographic measurements were made after 5 minutes of supine rest in a quiet and comfortable environment. After baseline examination, all subjects drank 168 mL/m^2^ (BSA, Gehan & George) of an energy drink containing caffeine (0.03%), taurine (0.4%), glucuronolactone (0.24%), glucose, and other ingredients, in maximum 5 minutes, and they underwent again echocardiography, electrocardiography, and blood pressure measurements 1 hour after drinking. Each participant was also studied in a control experiment by an equal volume of fruit juice one day after energy drink consumption. 

The analysis of files recorded was performed offline by a single, experienced, and independent echocardiographer, who did not know if the images refer to those obtained at baseline, after energy drink, or fruit juice consumption, using a commercially available, semiautomated, 2-dimensional strain software (EchoPac, GE, Milwaukee, WI, USA). The study protocol was in accordance with the Helsinki Declaration and the ethical standards of our institution, and all participants gave informed consent for participation in the study. 

### 2.3. Standard Echocardiography

Echocardiographic studies were performed using a high-quality ultrasound machine (Vivid 7; GE, Milwaukee, WI) with the subjects in the left lateral recumbent position. All measurements were made in accordance with current recommendations of American Society of Echocardiography (ASE) [12]. 

Left ventricle (LV) systolic function was analyzed by calculating left ventricle ejection fraction (LVEF), measured using Simpson's method, and by obtaining left ventricle longitudinal function parameters, as mitral annular systolic plane systolic excursion (MAPSE) with M-mode and mean peak systolic annular velocity with pulsed tissue-Doppler (mitral *S*′) by averaging values measured at septal and lateral mitral annulus [13]. Left ventricle diastolic function was assessed by the ratio between peak early (*E*) and late diastolic (*A*′) LV filling velocities with trans-mitral pulsed Doppler and by mean *E*′ and *A*′ mitral velocity with pulsed tissue Doppler [14]. *E*/*E*′ ratio was also calculated [15]. 

Right ventricle (RV) systolic function was assessed by calculating tricuspid annular plane systolic excursion (TAPSE) and mean peak systolic annular velocity by pulsed tissue Doppler on tricuspid annulus (tricuspid *S*′). 

### 2.4. Speckle-Tracking Echocardiography

The speckle-tracking analysis was performed in accordance with the indications of the ASE/EAE consensus document of Mor-Avi et al. [16]. Apical 4-chamber, 2-chamber, apical long-axis view, basal, and apical short axis view images were obtained using conventional 2-dimensional grayscale echocardiography, during breath hold and with a stable ECG trace. Three consecutive heart cycles were recorded and averaged. The frame rate was set between 60 and 80 frames per second. From apical views, we calculated left ventricular longitudinal strain (GLS), free wall and global right ventricular longitudinal strain (RVLS) [17], left atrial (LA), and right atrial (RA) longitudinal strain [18, 19]. For the Twisting analysis, after manual demarcation of LV endocardium by a point-and-click approach, at the basal and apical short-axis views, rotation-time curves were constructed for each segment. The global basal and apical rotations were estimated as the average angular displacement of 6 myocardial segments during systole. Rotation angles were expressed in degrees. LV twisting curve was automatically generated as the net difference between mean apical and basal rotation [20, 21]. 

### 2.5. Statistical Analysis

Data are reported as mean ± SD. Changes in the variables observed after ED and fruit juice consumption were compared using the Student's *t*-test for paired data. A *P* value <0.05 was considered statistically significant. Analyses were performed using the SPSS (Statistical Package for the Social Sciences, Chicago, IL) software release 11.5. 

## 3. Results

**Figure 1:**
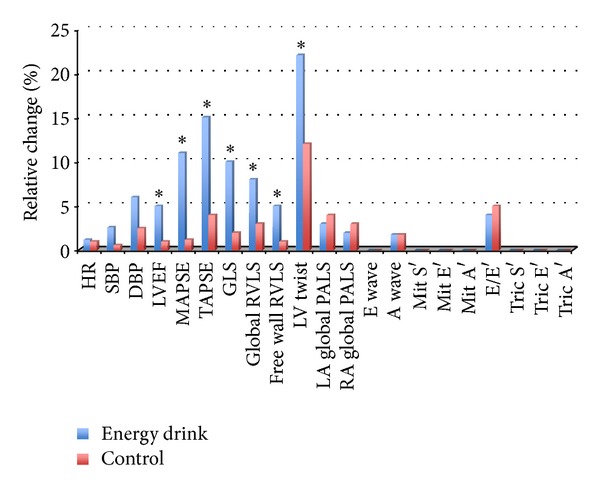
Mean relative increase from baseline. HR: heart rate; SBP: systolic blood pressure; DBP: diastolic blood pressure; LVEF: left ventricular ejection fraction; MAPSE: mitral annual plane systolic excursion; TAPSE: tricuspid annular plane systolic excursion; GLS: left ventricular global longitudinal strain; RVLS: right ventricular longitudinal strain; PALS: peak atrial longitudinal strain. *Significant versus control (fruit juice). For *P* value, see Table 2.

**Figure 2:**
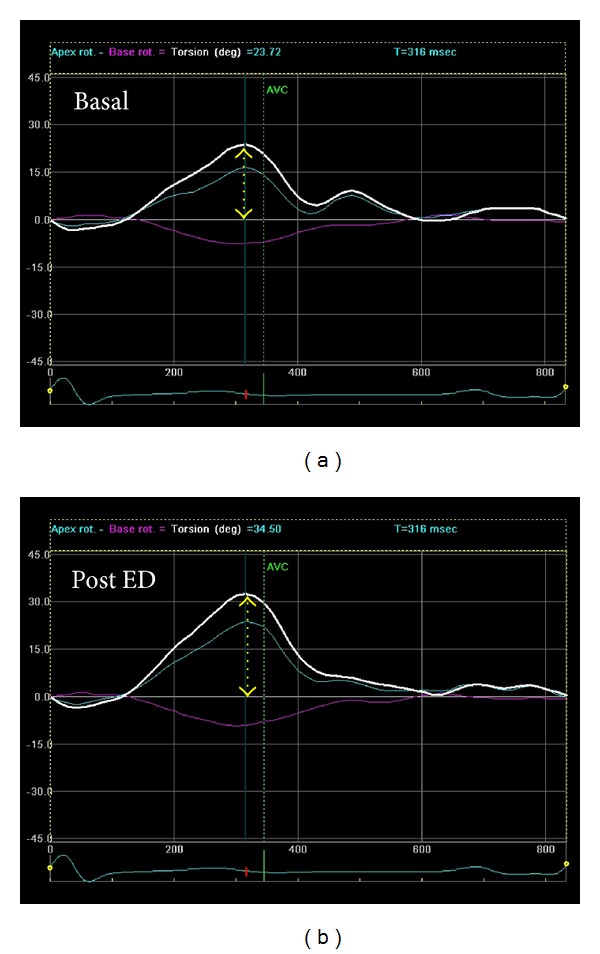
Left ventricular twisting at baseline and after energy drink (ED) consumption.

**Figure 3:**
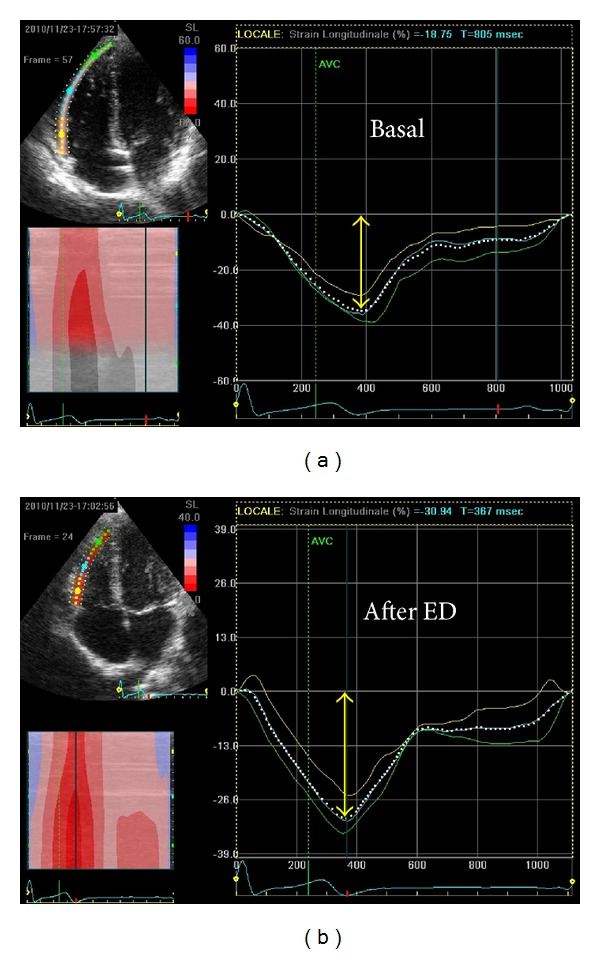
Free wall right ventricular longitudinal strain (RVLS) at baseline and after energy drink (ED) consumption.

All the variables at baseline and after taking ED are shown in Table 2. Figure 1 shows the mean relative increases of parameters studied at baseline, after energy drink, and in the control challenges. Significant variations occurred on LV myocardial deformation parameters after taking the energy drink (Figure 2). Mean relative increases of MAPSE, GLS, and twisting by STE were 11%, 10%, and 22% (Figure 1). All these variables had a very significant increase in respect to baseline with a *P* value of <0.001, 0.004, and <0.0001, respectively (Table 2). Mitral *S*′ did not show significant modification. LVEF showed an enhanced global LV systolic function with an increase of 5% (*P* = 0.01) from baseline. The parameters of RV deformation underwent significant changes: TAPSE, global RVLS, and free wall RVLS had a mean relative increase of 15% (*P* < 0.0001), 8% (*P* = 0.001), and 5% (*P* = 0.01), respectively (Table 2, Figure 3). Tricuspidal *S*′ did not show any modification. LA and RA deformation assessed by global peak of atrial longitudinal strain (PALS) did not undergo significant variations in respect to baseline. There were no significant changes in either Doppler parameters. Blood pressure and electrocardiographic parameters were showen to be roughly the same between groups, and a mild increase of 6% in diastolic blood pressure after ED consumption was not significant (*P* = 0.07). There were no significant changes in the parameters measured at baseline and after taking the juice, as shown in Table 3. 

## 4. Discussion

In addition to standard echo-Doppler analysis, speckle tracking echocardiography (STE) was used in our study to assess cardiac deformation in three spatial directions: longitudinal, radial, and circumferential. STE is a new technique for assessing myocardial function in physiological and pathological settings, and its feasibility and accuracy were tested in comparison with tagged magnetic resonance imaging, the gold standard to study myocardial deformation. It has been proved that STE is able to detect initial ventricular dysfunction in hypertension, diabetes, valvular heart disease, and heart failure, with high sensitivity in analyzing minimal change in myocardial deformation [11, 16]. 

In our study population of 35 young healthy subjects, taking an ED containing sugar, caffeine (0.03%), and taurine (0.4%) showed a significant change of myocardial function of both LV and RV one hour after drinking it, suggesting a possible positive effect on cardiac inotropism. 

In fact, the study of LV performance showed an increase of longitudinal function, with an increase of MAPSE and GLS, and a remarkable enhancement of LV Twisting (Figure 2). These modifications can probably explain the concomitant increase of LV global function represented by LVEF. 

Likewise, the study of RV performance showed an improvement of longitudinal function with a significant increase of TAPSE, and global and free RVLS in respect to baseline (Figure 3). Conversely, no significant changes in LA and RA function were found. Mitral and tricuspid plane excursion underwent a major change in respect to LV and RV longitudinal strain despite the higher sensitivity of the latter, but M-mode analysis only evaluates the displacement of the basal portion of the ventricles, unlike STE analysis that indicates the average of the displacements of all ventricular segments [11]. 

Such modifications on cardiac mechanics are probably due to the inotropic effect of some substances contained in the ED. 

In particular, taurine seems to have a similar digital effect by means of the control of myocardial contractility, modulating sarcoplasmic reticular Ca2+ release, and stimulating the pumping rate of Ca2+ activate ATPase pumps [8], preventing intracellular Ca2+ overload. 

In fact, it has been reported that taurine may reduce left ventricular end-diastolic pressure in patients with heart failure [22]. Furthermore, in Japan, taurine has been used clinically and has been claimed to be beneficial to patients that are unresponsive or resistant to digitalis or diuretics [23]. Also, the effect of caffeine could be involved in producing an increase in the myocardial contraction, but previous studies have ruled out a positive inotropic effect of caffeine on myocardial cells [24, 25]. An inotropic effect due to some substances of this ED can also explain the increase of LV twist after taking the drink. In fact, the effect of contractility on torsion has been well demonstrated; positive inotropic interventions, such as dobutamine infusion and paired pacing, greatly increase torsion [26–28], especially through augmentation of left ventricular apical endocardial rotation [29]. 

In normal subjects, LV twist represents a mechanism for generating stored energy during systole, which is released during early diastole to produce ventricular recoil, upward annular motion, and suction, confirming the close relation of systolic function to early diastole [30]. In normal hearts, all of these aspects of ventricular function increase on exercise to aid fast ejection and, more important, enable rapid filling of the ventricle during a shortened diastole period while maintaining a low filling pressure [31]. 

The LV twisting motion is a consequence of myocardial fiber orientation, which changes from an approximately longitudinal but slightly oblique orientation in the subendocardium to a circumferential orientation in the mid-wall and to an oblique orientation in the subepicardium [32, 33]. Thus, the subendocardial and subepicardial fibers represent two oppositely directed spirals and in normal heart because of larger radii, the torque of subepicardial fibers dominates over subendocardial fibers and accounts for the normal counterclockwise rotation of the LV apex.

Previous studies have found that in the setting of LV diastolic dysfunction, torsion is increased in patients with mild diastolic dysfunction but reduced in those with more severe degrees of diastolic dysfunction [34]; these changes may be explained by the fact that in the early stage, the subendocardial longitudinal fibers are primarily affected so that unopposed epicardial torque will increase apical twist, whereas later more widespread fibrosis or damage will affect global function and lead to reduced twist. 

Moreover, Tan et al. [31] showed that in patients affected by heart failure with preserved ejection fraction, in contrast with normal subjects, these abnormalities of LV function became more apparent on exercise, demonstrating a reduction of myocardial systolic strain, longitudinal function, and finally twisting motion. 

Since this study was performed on young healthy individuals at rest, future studies need to focus on whether such benefits persist after long term consumption of energy drinks and what the effects are of consuming these drinks during physical activity; it will also be important to determine which of the effects are induced in patients with cardiac disease to further our understanding of the potential benefits or risks of energy drink consumption. 

### 4.1. Study Limitations

Some limitations should be considered in this study. We evaluated young healthy subjects, so these results cannot be generalized for other populations. The number of subjects of our sample was limited, although it has allowed us to reach interesting conclusions. Moreover, we have investigated the acute changes on myocardial performance only one hour after energy drink consumption. Based on the pharmacokinetics of certain substances of this drink (such as caffeine and taurine), it should be interesting to evaluate the cardiovascular effect at different times from the consumption of these beverages. Lastly, we have used a pear juice as a control, but the same volume of this beverage does not correspond to the same amount of sugar dissolved in the energy drink.

## Figures and Tables

**Table 1:** Study population (*n* = 35).

Age	25 ± 2
Gender (% female)	17 (48.6%)
Weight (Kg)	65.8 ± 11
High (cm)	172.1 ± 8.2
Body surface area (m^2^)	1.78 ± 0.2
Drink (mL)	297.4 ± 1.9
Usual drinker (%)	8 (22.9%)
Current smoker (%)	10 (28.6%)
Physical activity (%)	6 (17%)

**Table 2:** Clinical and echocardiographic data after energy drink assumption (*n* = 35).

	Baseline	Energy drink	Relative change %	*P* value
HR (bpm)	67.1 ± 9.3	67.7 ± 9	1	0.69
SBP (mmHg)	118.2 ± 11.8	121.3 ± 10.3	3	0.35
DBP (mmHg)	77 ± 7.9	81.5 ± 9.3	6	0.07
LVEF (%)	63.2 ± 4.3	66.1 ± 4.5	5	0.01
Aortic CW (m/s)	1.14 ± 0.18	1.26 ± 0.17	9	0.01
MAPSE (mm)	15.6 ± 1.6	17.2 ± 1.8	11	<0.001
TAPSE (mm)	21.7 ± 2.9	25 ± 3.5	15	<0.0001
GLS (%)	−20.5 ± 2.2	−22.5 ± 3.2	10	0.004
LA global PALS (%)	42.5 ± 9.7	40.9 ± 6.5	3	0.41
RA global PALS (%)	44.3 ± 8.1	42.1 ± 9.3	2	0.54
Global RVLS (%)	−23.2 ± 3.8	−24.9 ± 3.9	8	0.001
Free wall RVLS (%)	−28.7 ± 5.9	−30.3 ± 6.1	5	0.01
LV twisting (degrees)	10.9 ± 4.1	13.5 ± 6.3	22	<0.0001
Mitral *E* (cm/s)	0.9 ± 0.1	0.9 ± 0.1	0	0.8
Mitral *A* (cm/s)	0.54 ± 0.1	0.53 ± 0.1	2	0.46
Mitral *S*′ (cm/s)	0.09 ± 0.02	0.09 ± 0.02	0	0.33
Mitral *E*′ (cm/s)	0.17 ± 0.02	0.17 ± 0.03	0	0.94
Mitral *A*′ (cm/s)	0.08 ± 0.02	0.08 ± 0.02	0	0.80
Mitral *E*/*E*′ (cm/s)	5.3 ± 1.05	5.07 ± 1.3	4	0.21
Tric *S*′ (cm/s)	0.13 ± 0.02	0.13 ± 0.01	0	0.71
Tric *E*′ (cm/s)	0.15 ± 0.03	0.16 ± 0.03	0	0.66
Tric *A*′ (cm/s)	0.1 ± 0.03	0.1 ± 0.02	0	0.88

HR: heart rate; SBP: systolic blood pressure; DBP: diastolic blood pressure; EF: ejection fraction; LV: left ventricle; RV: right ventricle; LA: left atrium; RA: right atrium; *E*: early transmitral flow velocity; *A*: atrial transmitral flow velocity; *S*′: systolic mitral annular velocity; *E*′: early diastolic mitral annular velocity; MAPSE: mitral annular plane systolic excursion; TAPSE: tricuspidal annulus plane systolic excursion; GLS: global longitudinal strain; RVLS: right ventricular longitudinal strain; PALS: peak atrial longitudinal strain.

**Table 3:** Clinical and echocardiographic data after fruit juice assumption (*n* = 35).

	Baseline	Fruit juice	Relative change %	*P* value
HR (bpm)	67.1 ± 9.3	67.8 ± 10.6	1	0.84
SBP (mmHg)	118.2 ± 11.8	117.5 ± 8.6	1	0.83
DBP (mmHg)	77 ± 7.9	75.0 ± 6.4	2	0.38
LVEF (%)	63.2 ± 4.3	63.8 ± 4.9	1	0.61
Aortic CW (m/s)	1.14 ± 0.18	1.16 ± 0.10	0	0.78
MAPSE (mm)	15.6 ± 1.6	15.3 ± 1.1	1	0.40
TAPSE (mm)	21.7 ± 2.9	23 ± 3.2	4	0.18
GLS (%)	−20.5 ± 2.2	−21.0 ± 1.9	2	0.36
LA global PALS (%)	42.5 ± 9.7	40.6 ± 8.6	4	0.52
RA global PALS (%)	44.3 ± 8.1	42.0 ± 9.3	3	0.70
Global RVLS (%)	−23.2 ± 3.8	−24.0 ± 3.2	3	0.53
Free wall RVLS (%)	−28.7 ± 5.9	−29.6 ± 4.9	1	0.60
LV twisting (degrees)	10.9 ± 4.1	9.7 ± 3.2	0	0.14
Mitral *E* (cm/s)	0.90 ± 0.10	0.90 ± 0.15	0	0.75
Mitral *A* (cm/s)	0.54 ± 0.10	0.53 ± 0.12	2	0.74
Mitral *S*′ (cm/s)	0.09 ± 0.02	0.09 ± 0.01	0	0.50
Mitral *E*′ (cm/s)	0.17 ± 0.02	0.16 ± 0.02	0	0.18
Mitral *A*′ (cm/s)	0.08 ± 0.02	0.07 ± 0.01	0	0.40
Mitral *E*/*E*′ (cm/s)	5.30 ± 1.05	5.55 ± 0.7	5	0.50
Tric *S*′ (cm/s)	0.13 ± 0.02	0.14 ± 0.02	0	0.74
Tric *E*′ (cm/s)	0.15 ± 0.03	0.15 ± 0.02	0	0.59
Tric *A*′ (cm/s)	0.1 ± 0.03	0.09 ± 0.01	0	0.74

HR: heart rate; SBP: systolic blood pressure; DBP: diastolic blood pressure; EF: ejection fraction; LV: left ventricle; RV: right ventricle; LA: left atrium; RA: right atrium; *E*: early transmitral flow velocity; *A*: atrial transmitral flow velocity; *S*′: systolic mitral annular velocity; *E*′: early diastolic mitral annular velocity; MAPSE: mitral annular plane systolic excursion; TAPSE: tricuspidal annulus plane systolic excursion; GLS: global longitudinal strain; RVLS: right ventricular longitudinal strain; PALS: peak atrial longitudinal strain.
